# The relationship between perceived stress and support with blood pressure in urban Haiti: A cross-sectional analysis

**DOI:** 10.1371/journal.pgph.0000263

**Published:** 2022-05-02

**Authors:** Lily D. Yan, Jessy G. Dévieux, Jean Lookens Pierre, Eliezer Dade, Rodney Sufra, Stephano St Preux, Olga Tymejczyk, Denis Nash, Miranda Metz, Myung Hee Lee, Dan W. Fitzgerald, Marie Deschamps, Jean W. Pape, Margaret L. McNairy, Vanessa Rouzier

**Affiliations:** 1 Department of Medicine, Division of General Internal Medicine, Weill Cornell Medicine, New York, New York, United States of America; 2 Department of Medicine, Center for Global Health, Weill Cornell Medicine, New York, New York, United States of America; 3 Department of Health Promotion and Disease Prevention, Robert Stempel College of Public Health and Social Work, Florida International University, Miami, FL, United States of America; 4 Haitian Group for the Study of Kaposi’s Sarcoma and Opportunistic Infections (GHESKIO), Port-au-Prince, Haiti; 5 City University of New York Institute for Implementation Science in Population Health, New York, NY, United States of America; Chiang Mai University, THAILAND

## Abstract

Haiti is a low-income country whose population lives under repeated and chronic stress from multiple natural disasters, civil unrest, and extreme poverty. Stress has been associated with cardiovascular (CVD) risk factors including hypertension, and the impact of stress on blood pressure may be moderated by support. The distribution of stress, support, and their association with blood pressure has not been well described in low-income countries. We measured stress and support using validated instruments on cross-sectional enrollment data of a population-based cohort of 2,817 adults living in Port-au-Prince, Haiti between March 2019 and April 2021. Stress was measured using the Perceived Stress Scale, while support was measured using the Multidimensional Scale of Perceived Social Support. Continuous scores were categorized into three groups for stress (low (1–5), moderate (6–10), high (11–16), and five groups for support (low (7–21), low-moderate (22–35), moderate (36–49), moderate-high (50–64), high (65–77)). Linear regression models were used to quantify the associations between: 1) support and stress adjusting for age and sex, and 2) stress and blood pressure adjusting for age and sex. A moderation analysis was conducted to assess if support moderated the relationship between stress and blood pressure. The cohort included 59.7% females and the median age was 40 years (IQR 28–55). The majority had an income <1 US dollar per day. The median stress score was moderate (8 out of 16 points, IQR 6–10), and median support score was moderate to high (61 out of 77 points, IQR 49–71). Stress was higher with older ages (60+ years versus 18–29 years: +0.79 points, 95% CI 0.51 to 1.08) and in females (+0.85 points, 95% CI +0.65 to +1.06). Support was higher in males (+3.29 points, 95% CI 2.19 to 4.39). Support was inversely associated with stress, adjusting for age and sex (-0.04 points per one unit increase in support, 95% CI -0.04 to -0.03). Stress was not associated with systolic or diastolic blood pressure after adjustment for age and sex. Support did not moderate the association between stress and blood pressure. In this urban cohort of Haitian adults living with chronic civil instability and extreme poverty, perceived levels of stress and social support were moderate and high, respectively. Contrary to prior literature, we did not find an association between stress and blood pressure. While support was associated with lower stress, it did not moderate the relationship between stress and blood pressure. Participants reported high levels of support, which may be an underutilized resource in reducing stress, potentially impacting health behaviors and outcomes.

## Introduction

Haiti has suffered a significant number of devastating natural disasters including the January 2010 earthquake in the West department, a cholera outbreak, the COVID-19 pandemic, the August 2021 earthquake in the Southern Peninsula, and recurrent political instability—all of which may act as prolonged sources of stress [1, 2]. High perceived stress from these conditions may contribute to the epidemic of cardiovascular disease (CVD), which is now the leading cause of death among adults in Haiti and many other low-middle income countries [3]. The association between perceived stress and poor health outcomes including elevated blood pressure, hypertension and cardiac dysfunction is well known from studies in high-income settings, but is not well studied in low-income settings [4–8].

Support from friends, family, and the surrounding community may help alleviate the impact of stress on individuals. In one common theoretical model, support acts as a buffer for stress at multiple points between a stressor and the negative physiological consequences [9]. Support may prevent the determination that an event is stressful, or it may intervene between the experience of stress and the onset of pathological outcomes by reducing stress reactions [9]. In another theoretical framework, higher support may also increase resources available to cope with stressful events, or assist in recovery [10]. Low job support has been associated with higher CVD risk, and lower well-being index [8]. Low social support has also been associated in longitudinal cohorts from high-income settings with increased risk of death (age adjusted relative risk of 2.3 for men, and 2.8 for women) [11].

Previous literature measuring and exploring stress, support, and CVD are almost exclusively based in high-income countries, without little to no evidence from lower income settings like Haiti. Haiti ranks 170 out of 189 countries by the United Nation’s Human Development Index, a combination measure that combines life expectancy, education, and standard of living [12]. Stressors in the form of recurrent natural and man-made disasters [1, 2] on top of chronic poverty and economic insecurity may be directly contributing to high rates of hypertension and CVD, which have only recently replaced infectious diseases as the most common causes of mortality [3, 13, 14]. While previous work has examined stress in Haiti, it has focused on specific events (post-earthquake) or small groups (adolescent girls) [15–17]. There remains a gap in systematic, population level measurement of this important health risk factor in lower-income settings like Haiti where instability is high.

The goal of this analysis is to estimate the prevalence of stress and support in a population-based cohort of adults in Port-au-Prince, Haiti and examine the relationship between stress, support, and the specific health outcome of blood pressure.

## Methods

### Study design and population

Data are from a cross-sectional survey within the Haiti CVD Cohort Study (clinicaltrials.gov NCT03892265), which follows a population-based cohort of Port-au-Prince residents selected using multistage random sampling. Inclusion criteria were age ≥ 18 years, primary residence in Port-au-Prince, and absence of any serious medical condition or cognitive impairment preventing participation, as previously described [18]. This larger study aims to enroll 3000 participants without cardiovascular disease and follow them for 2 to 3.5 years to evaluate the prevalence and incidence of CVD risk factors and diseases, such as hypertension, diabetes, obesity, dyslipidemia, kidney disease, poor diet, smoking, physical inactivity, and inflammation. The current analysis includes participants enrolled between March 19, 2019 and April 30, 2021 (n = 2847). Participants missing information on age, sex, perceived stress, perceived support, or blood pressure measurement (n = 30) were excluded from our analysis (Fig A in S2 Text).

The study was conducted at the Groupe Haitien d’Etude du Sarcome de Kaposi et des Infections Opportunistes clinics (GHESKIO), a medical non-profit organization that has operated continuously over four decades in Haiti to provide clinical care and conduct research on HIV and related infections and chronic diseases.

### Measurements and scales

Stress, support, and BP measurements were collected from participants at the time of study enrollment.

Stress was measured through the Perceived Stress Scale 4 (PSS-4) [19], a shortened 4-question version of the original 14 question Perceived Stress Scale [20]. Answer choices range from 0 = Never to 4 = Very Often. Although the PSS-4 has moderate loss in internal reliability versus the PSS-14, the PSS-4 is commonly used in settings with time constraints [19]. We used all questions from the PSS-4 translated into Haitian Creole, with a total score calculated by tabulating individual questions, ranging from 0 to 16. Cronbach’s alpha was 0.37. For comparability to the World Health Survey that uses a two question version of the PSS [21], we also calculated the PSS-2 from the first two questions, with a total score ranging from 0 to 8.

Support was measured through an adapted Multidimensional Scale of Perceived Social Support (MSPSS), with eleven questions and seven possible answer choices (1 = Very strongly disagree to 7 = Very strongly agree), for a total number of points ranging from 7 to 77 [22]. Questions were translated into Haitian Creole. MSPSS had a Cronbach’s alpha of 0.87 in our sample. Table A in S2 Text lists the questions in English and Creole for the PSS-4 and the MSPSS used in this study.

Blood pressure (BP) measurements were taken following World Health Organization and American Heart Association guidelines [23, 24], using semi-automated electronic cuffs (OMRON HEM 907). After resting for five minutes, the participant had three BPs measured, separated by 1-minute intervals. The average of the last 2 BPs was the BP used for analysis. Systolic blood pressure (SBP) and diastolic blood pressure (DBP) were considered separately in this analysis.

### Statistical analysis

In descriptive analyses, total scores for stress and support were summarized both as continuous variables (mean, standard deviation, median, interquartile range (IQR: 25^th^ to 75^th^ percentiles)), and as categorical variables for interpretability. Stress was categorized as low (1–5), moderate (6–10), high (11–16) to make equal-interval groups. Support was categorized as low (7–21), low-moderate (22–35), moderate (36–49), moderate-high (50–64), high (65–77)) to make equal-interval groups. BP was summarized as a continuous variable. Age was divided into categories based on the hypothesis that older ages (60 years and above) might have a different association than younger ages (18–29 years) with outcomes like stress, support, and BP. Bar charts were generated to visualize stress and support distribution in the overall sample, and by sex.

For inferential analyses, we first examined the association of age and sex with either stress or support using linear multivariable regressions. We then examined the association between support and stress adjusting for age and sex. Finally, we examined the association between stress and BP, adjusting for age and sex, and performed a moderation analysis of support on the association between stress and BP, adjusting for age and sex where the interaction term stress*support was the term of interest.

Age was categorical for the initial analyses with stress and support as outcomes, and continuous for models with BP as outcomes to facilitate moderation analysis. All outcomes (stress, support, SBP, DBP) were continuous. In moderation analysis, stress and support were centered (i.e., the overall sample mean score was subtracted from the individual participant score), so that the coefficient of stress or support could be interpreted as the effect of that variable on SBP or DBP at the mean level of the other independent variable.

All analyses were conducted in R, version 4.0.3 [25].

### Ethics

This study was approved by institutional review boards at Weill Cornell Medicine and GHESKIO. Written informed consent was obtained from all participants.

## Results

Out of a total sample size of 2,817 adults, 59.7% were female and the median age was 40 years. A third of participants had no education or a primary level (36.2%) and the majority had an employment income of less than 1 US dollar a day (69.7%) (Table 1).

**Table 1:** Characteristics of Haiti CVD cohort study.

	Haitian cohort (N = 2817)
	N or median (% or IQR)
**Female**	1682 (59.7)
**Age**, y	
Median [IQR]	40 [28, 55]
Mean (SD)	42.0 (16.0)
18–29	825 (29.3)
30–39	534 (19.0)
40–49	491 (17.4)
50–59	465 (16.5)
60+	502 (17.8)
**Education**	
Primary or lower	1019 (36.2)
Secondary or higher	1798 (63.8)
**Employment Income (daily)**	
≤1 USD	1963 (69.7)
>1 USD	854 (30.3)
**Perceived Stress Scale**	
Range	0–16
Median [IQR]	8 [6, 10]
Mean (SD)	7.85 (2.72)
Low (1–5)	525 (18.6)
Moderate (6–10)	1898 (67.4)
High (11–16)	394 (14.0)
**Multidimensional scale of Perceived Support**	
Range	7–77
Median [IQR]	61 [49, 71]
Mean (SD)	58.7 (14.7)
Low (7–21)	44 (1.6)
Low-Moderate (22–35)	177 (6.3)
Moderate (36–49)	522 (18.5)
Moderate-High (50–64)	863 (30.6)
High (65–77)	1211 (43.0)
**Systolic Blood Pressure**	
Median [IQR]	118 [106.5, 138.0]
Mean (SD)	124.1 (24.0)
**Diastolic Blood Pressure**	
Median [IQR]	72.0 [62.5, 83.5]
Mean (SD)	73.9 (15.6)

### Stress

**Fig 1:**
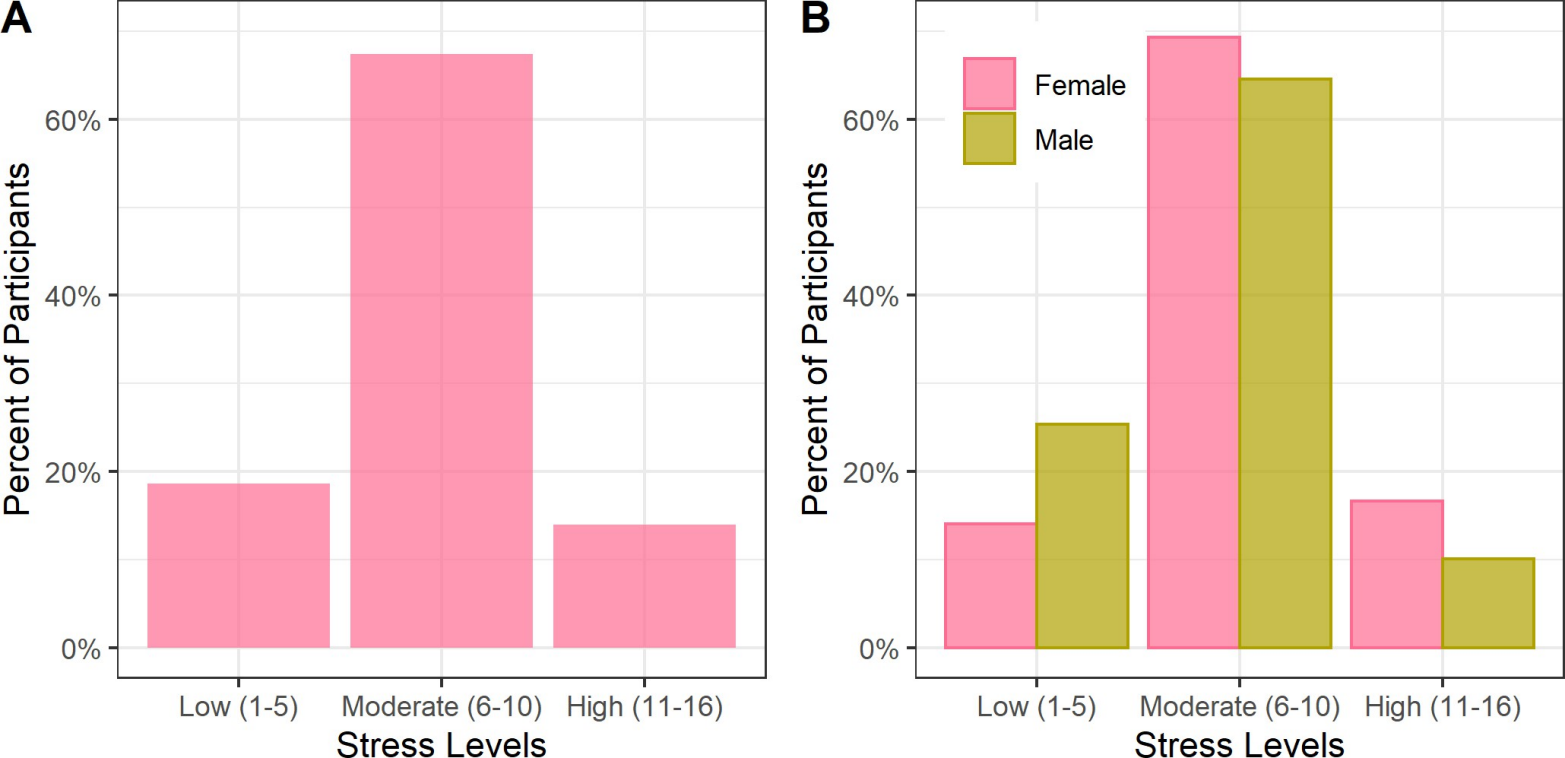
Moderate perceived stress among adult cohort in Haiti (N = 2817). Legend: The percent of participants in each stress level is shown in Panel A. The percent of participants by sex in each stress level is shown in Panel B. Moderate levels of stress were seen overall, with lower stress in men versus women.

The median PSS score was 8 (IQR 6–10), with 18.6% of participants having low stress, 67.4% moderate stress, and 14.0% high stress (Fig 1A). Males had lower stress scores (Fig 1B). Stress was higher among older participants and in females (Table B in S2 Text). Using the methodology in the World Health Surveys, the mean PSS-2 score was 6.0 (min 2, max 10).

In linear regression on stress with age and sex, compared to people aged 18–29 years, older age categories were associated with higher stress (40–49: 0.47 points, 95% CI 0.17 to 0.77; 60+: 0.79 points, 95% CI 0.51 to 1.08). Males reported less stress than females (-0.85 points, 95% CI -1.06 to -0.65).

### Support

**Fig 2:**
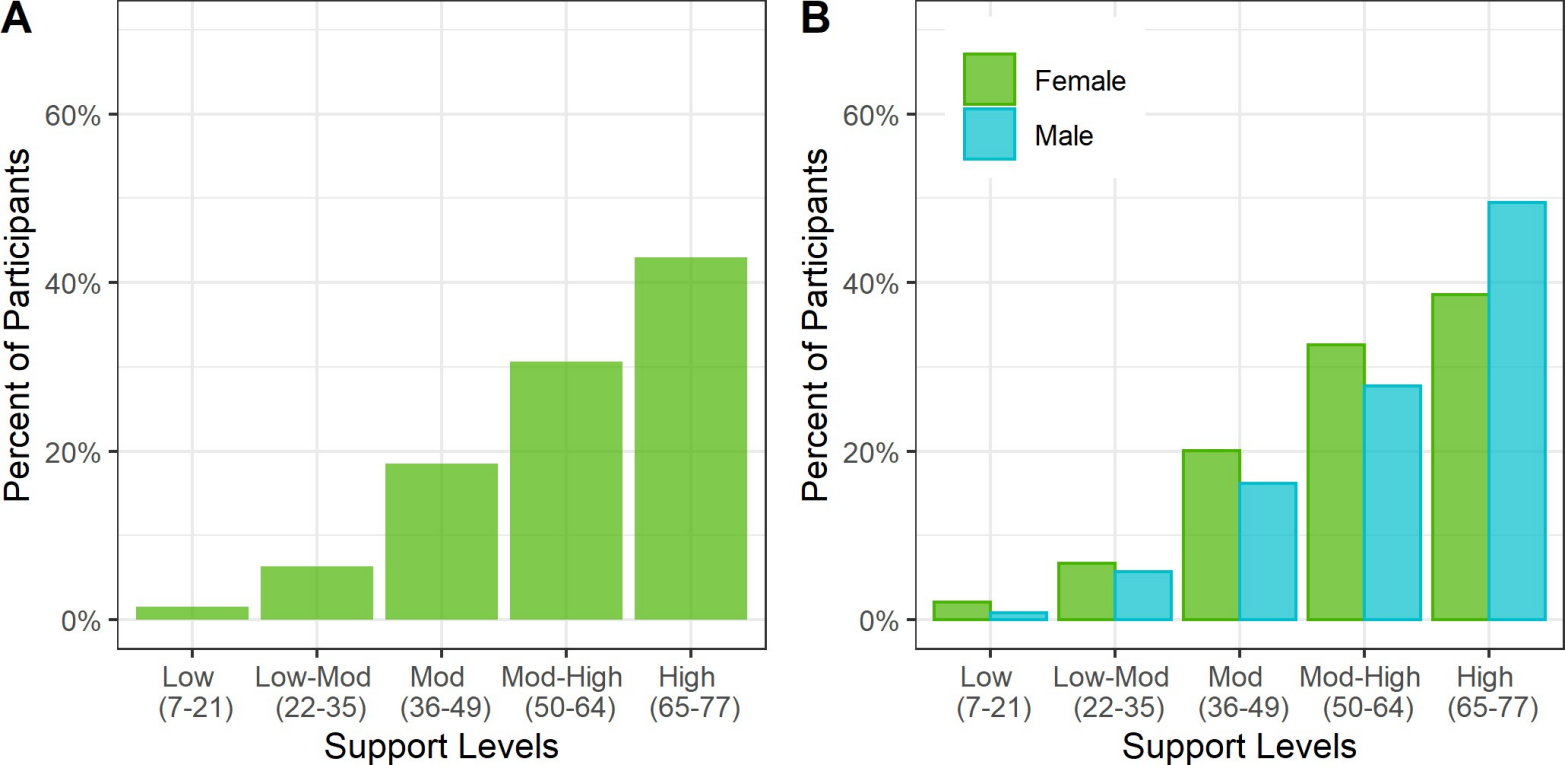
High perceived support among adult cohort in Haiti (N = 2817). Legend: The percent of participants in each support level is shown in Panel A. The percent of participants by sex in each support level is shown in Panel B. High levels of support were seen overall, with higher levels of support in men versus women.

The median MPSS score was moderate-high at 61 (IQR 49–71). The distribution of support showed a left skew, which was more prominent for men than women (Fig 2A and 2B).

In linear regression on support with age and sex, compared to people aged 18–29 years, older age categories were associated with lower support (30–39: -2.08 points, 95% CI -3.65 to -0.5; 40–49: -2.29 points, 95% CI -3.93 to -0.65; 50–59: -1.98 points, 95% CI -3.65 to -0.32). Men had significantly higher support than women (3.29 points, 95% CI 2.19 to 4.39).

In examining the relationship between stress and support, after adjusting for age and sex, participants with a 1-point higher support level had a -0.04 point lower stress level (95% CI -0.04 to -0.03).

### Associations between stress, support, and blood pressure

In unadjusted analysis, stress was associated with higher SBP (0.43 mmHg, 95% CI 0.12 to 0.74) and higher DBP (0.42 mmHg, 95% CI 0.21 to 0.62). However, stress level was not significantly associated with SBP or DBP after adjusting for age and sex (Table 2, Model 1–2). In moderation analysis, the interaction term of stress*support was not statistically significantly associated with either SBP or DBP, suggesting support does not moderate the relationship between stress and BP in our sample (Table 2, Models 3–4).

**Table 2:** Association of stress, support, systolic blood pressure and diastolic blood pressure by linear regression.

	Model 1				Model 2				Model 3				Model 4			
	SBP				DBP				SBP				DBP			
	β	95% CI		p value	β	95% CI		p value	β	95% CI		p value	β	95% CI		p value
Stress*	-0.02	-0.28	0.23	0.86	0.07	-0.11	0.26	0.43	0.01	-0.25	0.27	0.94	0.09	-0.1	0.28	0.34
Age	0.87	0.82	0.92	<0.01	0.45	0.41	0.48	<0.01	0.87	0.82	0.92	<0.01	0.45	0.41	0.48	<0.01
Male vs Female	3.95	2.47	5.43	<0.01	-1.94	-3	-0.88	<0.01	3.89	2.4	5.38	<0.01	-1.96	-3.02	-0.9	<0.01
Support*	--				--				0.03	-0.02	0.08	0.3	0.01	-0.02	0.05	0.52
Stress x Support*	--				--				0.004	-0.01	0.02	0.63	0.006	-0.006	0.02	0.38
Intercept	86.1				55.4				86				56			
Sample Size	2817				2817				2817				2817			

* Stress and Support have been centered to aid in interpretation, ie one unit change is from the mean level of stress or support

## Discussion

Despite a challenging and volatile political context, coupled with severe economic deterioration, urban Haitian adults reported moderate levels of perceived stress coupled with high levels of perceived support. Higher perceived stress was more common in older age groups, and in women compared to men in Haiti. Higher levels of perceived support were more common in men compared to women. Stress was not associated with BP, and support did not moderate this relationship.

Haiti is a unique context to measure perceived stress, as it has had a persistent and longstanding history of civil unrest since it gained independence from France in 1804. In the intervening 200 years, Haiti has had 22 constitutions and 43 heads of state, of which 30 were assassinated or overthrown, the last as recently as July 2021 [2]. In modern times, the locus of violence has shifted towards more diffuse actors including paramilitary groups and gangs [2]. Moreover, Haiti has faced recurrent natural disasters, including the 2010 earthquake estimated to have killed 250,000, the cholera outbreak killing over 10,000, recurrent hurricanes, the COVID-19 pandemic, and the most recent August 2021 earthquake in the Southern Peninsula responsible for an estimated 2,207 dead and over 136,000 destroyed buildings [1]. This contributes to an environment of persistent economic instability for many Haitians who live with extreme poverty and food insecurity [13].

Despite severe poverty and persistent insecurity, our cohort reported lower than anticipated perceived stress from such a sociopolitical context. One explanation could be desensitization to political, economic, and civil instability with prolonged exposure. Another is that some of the PSS-4 questions, especially the question related to lack of control, do not adequately capture stress in the Haitian context. This may be reflected by the low Cronbach’s alpha in our sample for the PSS-4. The social workers and study physicians reflect that stress in Haiti often manifests as psychosomatic symptoms: back aches, head aches, pain in the body, and a rapid heart rate—which are not captured in the PSS-4. Furthermore, there may have been some stigma against reporting stress. One social worker suggested that participants may not have wanted to report the details of their struggles as a protective mechanism, since verbalizing the struggles would potentially allow their circumstances to destroy them. In Haitian Creole this was expressed as “*yo pa vle plonje*, *yo pa vle efondre*, *yo fé aktivite yo kan menm”*, or they do not want to dive, they do not want to collapse, so they continue to carry on. Finally, reported stress may not reflect an individual’s resilience and ingenuity in coping with stress through spiritual, mental, or emotional practices [26].

The measurement of stress and specific scales used has varied in other low-middle income countries (LMICs), making direct comparison of our findings to similar contexts challenging. Indirect comparisons suggest stress levels are chronic and higher in Haiti compared to other LMICs and high-income countries. A meta-analysis of mental health after the 2010 earthquake in Haiti reported 28% of individuals had severe symptoms of post-traumatic stress disorder, and 20% had severe symptoms of anxiety [15]. Our findings that 14% of participants had high levels of stress and 81.4% had at least moderate levels of stress suggest that these symptoms are chronic and have persisted beyond the acute post-earthquake period. One of the largest surveys of stress across LMICs is the World Health Survey (WHS), a community based study from 2002–2004 of 70+ countries that incorporated a two question version of the PSS with a Likert scale scored from 1 to 5, reporting a mean PSS-2 stress score of 2.8 globally, 5.1 in Africa and 3.7 in the Americas [21]. If we analyze our stress scores using the WHS methodology, we find a mean score of 6.03 (min 2, max 10), which is higher than countries in Africa or the Americas [21].

The sex breakdown of higher stress in women versus men is consistent with prior literature [19, 27], with multiple explanatory theories including the differential exposure hypothesis that women experience more stressors from societal expectations and sex-based discrimination [28]. Our finding that older participants report higher stress versus younger participants is discordant with prior research, which reports lower stress in older people in high income settings [29]. One explanation for this age difference in stress is that older people in high income settings have fewer responsibilities in retirement after child-rearing is over, compared to younger people, and older age may be associated with advantages in behaviors related to emotional regulation [19, 29]. This predictable life-course may not be true in LMICs with substantial civil unrest like Haiti. First, familial stressors may not be reduced with older age, if older participants take on child rearing roles of younger relatives due to the expense of childcare or after the death of parents. Second, there may not be “retirement” for older participants living in urban Port-au-Prince, given the lack of economic support or pension plans, requiring them to continue working.

Support was high in our sample, notably much higher in men compared to women. This is a finding which has been found repeatedly in high income cohorts, as well as in an adolescent cohort in Port-au-Prince [17, 30]. While women have reported more close personal relationships within their social groups, men have larger social networks, possibly reflecting different patterns of socialization with women more likely to form confidantes, and men more likely to move within groups [30]. Differences in societal expectations of caregiving may also play a role in why women perceive lower support [31]. Women are also more likely to provide emotional support than men at both home and at work, suggesting women may be the primary providers of emotional support rather than the primary recipients [32].

Contrary to prior research, we did not find an association between stress and blood pressure [33]. A prior meta-analysis found increased stress was associated with an increased risk of hypertension (OR 2.40), and hypertensive patients had higher incidence of psychosocial stress (OR 2.69) [33]. Our negative finding could have resulted from a relative lack of variation in reported stress. Or, perhaps other risk factors such as age dominate the development of hypertension in Haiti. If the PSS-4 did not accurately capture stress levels in Haiti due to different cultural expressions of stress as detailed above, that may also have contributed to this lack of association. Lastly, our sample size is smaller than the pooled samples found in meta-analyses that have demonstrated a relationship between stress and hypertension. Given this first negative finding, unsurprisingly we did not find that support moderated the relationship between stress and blood pressure.

It is unclear if support may have other positive impacts on CVD-related health. For example, support has been associated with better health behaviors, including greater odds of physical activity and adequate fruit-vegetable intake of 5 portions per day [34]. Furthermore, in Ethiopia among people with hypertension, good support was associated with good hypertension self-care practices including self-reported diet and exercise [35]. Future interventions to capitalize on support may include strengthening support systems within existing infrastructure of family or religious organizations to promote healthy diets and exercise.

This study’s strengths include the large, population-based sample in a rarely-described urban, low-income country setting. The research-grade instruments used for blood pressure also allows analysis of accurate measurements. Limitations include self-reported cross-sectional data, low Cronbach’s alpha for the PSS-4, the use of potentially artificial equal-interval groupings for stress and support, and potential selection bias due to civil unrest—weeks with higher civil unrest may have resulted in selection bias for those able or willing to participate in the study, which may have resulted in biased reporting of stress and support.

In summary, Haitian adults living in Port-au-Prince reported moderate levels of perceived stress, coupled with high levels of support. Stress was not associated with BP. Higher support was associated with lower stress, however, support did not moderate the effect of stress on blood pressure. These findings are notable given the extreme civil and economic stressors facing Haitians and may reflect the long record of resilience and ingenuity among this remarkable population of survivors. Future research should explore and leverage existing structures of support, which may be an underutilized resource in reducing stress, potentially impacting health behaviors and outcomes.

## Supporting information

S1 Text (DOCX)

S2 Text (DOCX)
