# Supplementary material for: The relationship between perceived stress and support with blood pressure in urban Haiti: A cross-sectional analysis
Source: PLOS Glob Public Health. 2022 May 2;2(5):e0000263. doi: 10.1371/journal.pgph.0000263 (PMC9249088; doi:10.1371/journal.pgph.0000263)
Supplement: S1 Text — (DOCX) [file pgph.0000263.s001.docx]

Inclusivity in global research

PLOS’ policy on inclusivity in global research aims to improve transparency in the reporting of research performed outside of researchers’ own country or community and ensures that PLOS publications reporting global research adhere to high standards for research ethics and authorship. Authors of relevant research articles may be asked to complete the questionnaire below, which outlines ethical, cultural, and scientific considerations specific to inclusivity in global research. This questionnaire may be requested when researchers have travelled to a different country to conduct research, if research uses samples collected in another country, research with Indigenous populations or their lands, or if research is on cultural artefacts. Researchers travelling to another country solely to use laboratory equipment will not normally be required to complete the questionnaire. However, the questionnaire can be requested at the journal’s discretion for any submission – if you have been requested to complete this questionnaire by the PLOS journal you submitted to, please do so.

Please complete the questionnaire below and include this as a Supporting Information file with your manuscript. Note that if your paper is accepted for publication, this checklist will be published with your article in the supporting information files. Please ensure that you reference the checklist in the main body of your manuscript. We suggest adding a subsection ‘Inclusivity in global research’ to your Methods section and adding the following sentence: “Additional information regarding the ethical, cultural, and scientific considerations specific to inclusivity in global research is included in the Supporting Information (SX Checklist)”

The questions have been designed to be applicable to a wide range of study types, and there are subsections for both human subjects research and non-human subjects research. If any of the questions are not relevant to your research please mark them as “N/A” as appropriate.

**Ethical considerations, permits and authorship**

*This section is applicable to all research types.*

Provide details as to who granted permissions and/or consent for the study to take place in the Methods section of your manuscript. This should include the names of **all** ethics boards, governmental organizations, community leaders or other bodies that provided approval for the study. If individuals provided approval refer to these people by their role or title but do not list their name(s).

Reported on page number: 9

If there were any deviations from the study protocol after approval was obtained please provide details of these changes in the Methods section of your manuscript.
Did this study involve local collaborators that are residents of the country where the research was conducted or members of the community studied? If you do not have any authors from said communities, please provide an explanation for this below.

Reported on page number: There were no deviations from the study protocol after approval and consent was obtained.

Yes, this research involved local collaborators that are residents of Haiti. Most of the coauthors are from Haiti (JL Pierre, E Dade, R Sufra, S St Preux, M Deschamps, J Pape, and V Rouzier)

Everyone listed as an author should meet PLOS’ criteria for authorship and all individuals who meet these criteria should be included in the author byline, rather than the acknowledgements. Authorship criteria is based on the International Committee of Medical Journal Editors (ICMJE) Uniform Requirements for Manuscripts Submitted to Biomedical Journals - for further information please see here: <https://journals.plos.org/plosone/s/authorship>.

**Human subjects research (e.g. health research, medical research, cross-cultural psychology)**

Did you obtain written informed consent from a representative of the local community or region before the research took place? How did you establish who speaks for the community? Details of written informed consent obtained from study participants should be reported separately in the Methods section of your manuscript.

Written informed consent was obtained from all research participants. Ethics review and approval was obtained for the study from the GHESKIO Centers Ethics Committee that oversees all research involving participants seen at GHESKIO Centers. All research projects at GHESKIO are also presented to the Community Advisory Board for input. The Community Advisory Board consists of representatives from the civil society including people living with HIV, illiterate people, women’s group leader, religious leaders, doctors, lawyers, students, teachers etc. Research staff work closely with the GHESKIO Community Advisory Board and are sensitized to conducting community-based research.

How did members of the local community provide input on the aims of the research investigation, its methodology, and its anticipated outcome(s)?

Members of the local community provide input on research projects primarily through the Community Advisory Board meetings. During these sessions, presentations are made by the GHESKIO research team to the CAB, and through open discussion feedback on the aims, the methodology, and outcomes are elicited.

When engaging with the local community, how did you ensure that the informed consent documents and other materials could be understood by local stakeholders?

GHESKIO is committed to the ethical conduct of research following the international standards of the Helsinki Declaration and US Federal Registry. In 1983, GHESKIO established an IRB responsible for reviewing the ethics of research protocols. The IRB has United States Federal Wide Assurance and is registered with the Office for Human Research Protections. The informed consent forms and all other patient materials are translated in Haitian Creole and reviewed and corrected by the GHESKIO IRB prior to approval and implementation at the site.

GHESKIO has established procedures to assure the voluntary informed consent of research participants. Once identified as a potential participant, study staff provide the potential participant information regarding the study including: voluntary nature of trial participation, delivery of free care at GHESKIO regardless of study participation, all study procedures, number of visits, risks and benefits of study participation, and alternatives to study participation. Ample time is given to each volunteer to discuss the study and to ask and receive answers to all questions and concerns. Social workers go over the Informed Consent Form (ICF), and Assent Form for minors (when applicable), with the potential participant or parent(s)/guardian(s) in their native language (Haitian Creole). They have the opportunity to discuss the ICF and study participation further with their families. If after this discussion, the potential participant is willing to proceed, they complete an Assessment of Understanding quiz. Participants must receive at least 80% on the quiz to continue. Once understanding of the study has been verified, the participant or parent(s)/guardian(s) will sign the Informed Consent Form, and any minor the Assent Form. Each volunteer is given a copy of the signed consent for their records.

The Consent process and consent forms are in Creole.

Will the findings of the research be made available in an understandable format to stakeholders in the community where the study was conducted (e.g. via a presentation, summary report, copies of publications, etc.)? Please provide details of how this will be achieved.

Findings of the research for the larger Haiti CVD Cohort Study will be converted into a powerpoint presentation and shared with the Community Advisory Board. Copies of the publication will be shared with other stakeholders, such as interested members of the Ministry of Health, the Haitian College of Cardiology, and other healthcare providers at GHESKIO.

**Non-human subjects research using specimens/ animals collected as part of the study, or those housed in archival collections. Examples include archaeology, paleontology, botany and zoology.**

Did the permission you obtained from a local authority to perform the study include an agreement on access to outputs and benefit sharing? This may include procedures to enable fair distribution of the benefits and resources arising from the research performed. Please include any details of Prior Informed Consent and Benefit Sharing Agreements obtained. These may be required by field-specific regulations, for example the Convention on Biological Diversity (CBD) and the associated Nagoya Protocol.

N/A

If the material used in your study was imported, please A) provide the year it was imported and B) indicate whether permits were obtained to import/export the materials used, C) provide details of any permits obtained. If this information is not available, please indicate this.

N/A

If you used archival specimens, please state how the material used in your study was acquired by the institute it is held in and provide details of any permits obtained for the original excavations/ sample collection. If this information is not available, please indicate this.

N/A

How was the potential cultural significance of the materials collected in your study to local communities considered in your research design? Were Indigenous peoples and/or local researchers and institutions involved with archaeological excavations / collection of specimens? If so, please provide a description of their involvement.

N/A

If your manuscript includes photographs of human remains please indicate whether authors obtained permission from descendants or affiliated cultural communities to do so.

N/A
