# Supplementary material for: The relationship between perceived stress and support with blood pressure in urban Haiti: A cross-sectional analysis
Source: PLOS Glob Public Health. 2022 May 2;2(5):e0000263. doi: 10.1371/journal.pgph.0000263 (PMC9249088; doi:10.1371/journal.pgph.0000263)
Supplement: S2 Text — (DOCX) [file pgph.0000263.s002.docx]

**Online Supplementary File**

**Title:** Perceived stress, social support, and blood pressure in urban Haiti

**Authors**: Lily D. Yan^1,2^, Jessy G Dévieux^3^, Jean Lookens Pierre^4^, Eliezer Dade^4^, Rodney Sufra^4^, Stephano St Preux^4^, Olga Tymejczyk^5^, Denis Nash^5^, Miranda Metz^1,2^, Myung Hee Lee^2^, Dan W. Fitzgerald^2^, Marie Deschamps^4^, Jean W. Pape^2,4^, Margaret L. McNairy ^1,2,co^, Vanessa Rouzier ^2,4,co^

**Affiliations**

1. Division of General Internal Medicine, Department of Medicine, Weill Cornell Medicine, New York, New York, USA

2. Center for Global Health, Department of Medicine, Weill Cornell Medicine, New York, New York, USA

3. Department of Health Promotion and Disease Prevention, Robert Stempel College of Public Health and Social Work, Florida International University, Miami, FL, USA.

4. Haitian Group for the Study of Kaposi's Sarcoma and Opportunistic Infections (GHESKIO), Port-au-Prince, Haiti

5. City University of New York Institute for Implementation Science in Population Health, New York, NY, USA

**co**: contributed equally to this work

**Corresponding author:**

Lily D Yan, MD MSc

Instructor of Medicine

Center for Global Health and Division of General Internal Medicine

Weill Cornell Medicine

402 East 67th Street, 2nd floor

NY, NY 10065

Telephone: (646) 962-8140, Fax: (646) 962-0285

[liy9032@med.cornell.edu](mailto:liy9032@med.cornell.edu)

**Table A: Perceived stress scale and perceived support scale translations**

| **Perceived Stress Scale**  Adapted from Cohen 1983 | **Haitian Creole Translation** | **Answers** | **Haitian Creole Translation** |
| --- | --- | --- | --- |
| In the past month, I felt a lack of control | Nan dènye mwa ki sot pase a, konbyen fwa ou te santi ou pat kapab kontwole bagay enpòtan nan lavi ou? | Never (0),  Almost never,  Sometimes,  Fairly often,  Very often (4) | Jamè (0),  Prèske jamè,  Pafwa,  Byen souvan,  Trè souvan (4) |
| In the past month, I felt confident that I can manage my personal problems | Nan dènye mwa ki sot pase a, konbyen fwa ou te santi ou gen konfyans sou kapasite w pou jere pwoblèm pèsonèl ou yo? |  |  |
| In the past month, things went my way | Nan dènye mwa ki sot pase a, konbyen fwa ou te santi bagay yo te ale jan ou ta renmen an? |  |  |
| In the past month, I felt my difficulties were piled too high to overcome | Nan dènye mwa ki sot pase a, konbyen fwa ou te santi difikilte yo te anpile tèlman wo ou pa t kapab sipote yo? |  |  |
| **Perceived Support Scale**  Adapted from Zimet 1988 | **Haitian Creole Translation** | **Answers** | **Haitian Creole Translation** |
| There is a special person who is around when I am in need | Mwen gen yon moun espesyal mwen ka jwenn fasilman lè mwen bezwen. | Very strongly disagree (1),  Strongly disagree,  Mildly disagree,  Neutral,  Mildly agree,  Strongly agree,  Very strongly agree (7) | Pa trè dakò nèt (1),  Pa dakò nèt,  Pa dakò,  Ni dakò ni pa dakò,  Byen dakò,  Dakò dakò,  Trè dakò nèt (7) |
| There is a special person with whom I can share my joys and sorrows | Mwen Gen yon moun espesyal pou mwen pataje kontantman ak lapènn mwen. |  |  |
| My family really tries to help me | Fanmi mwen vrèman ap eseye ede m. |  |  |
| I get emotional help and support I need from my family | Mwen jwenn èd emosyonèl ak sipò mwen bezwen nan fanmi mwen. |  |  |
| I have a special person who is a real source of comfort to me | Mwen gen yon moun espesyal ki se yon sous pou rekonfòtem. |  |  |
| My friends really try to help me | Zanmi mwen vrèman eseye ede m. |  |  |
| I can talk about my problems with my family | Mwen ka pale sou pwoblèm mwen ak fanmi mwen. |  |  |
| I have friends with whom I can share my joys and sorrows | Mwen gen zanmi pou mwen pataje kontantman ak lapènn mwen |  |  |
| There is a special person in my life who cares about my feelings | Gen yon moun espesyal nan lavi mwen ki okipe de santiman mwen. |  |  |
| My family is willing to help me make decisions | Fanmi mwen an vle ede m pran desizyon. |  |  |
| I can talk about my problems with my friends | Mwen ka pale avek zanmi mwen sou pwoblèm mwen yo. |  |  |

**Table B: Perceived stress and support levels among Haiti Cardiovascular Disease Cohort (n = 2817)**

|  |  | **Stress** | | **Support** | |
| --- | --- | --- | --- | --- | --- |
|  | **N** | **Mean ± SD** | **Median (IQR)** | **Mean ± SD** | **Median (IQR)** |
| **Total** | **2817** | 7.85 ± 2.72 | 8 (6, 10) | 58.7 ± 14.7 | 61 (49, 71) |
| **Age Category** |  |  |  |  |  |
| 18-29 | 825 | 7.50 ± 2.83 | 8 (6, 9) | 60.3 ± 13.5 | 63 (51, 71) |
| 30-39 | 534 | 7.73 ± 2.89 | 8 (6, 10) | 57.8 ± 15.1 | 59 (48, 70.75) |
| 40-49 | 491 | 8.08 ± 2.61 | 8 (7, 10) | 57.6 ± 15.4 | 59 (47, 71) |
| 50-59 | 465 | 7.83 ± 2.65 | 8 (6, 10) | 57.9 ± 15.4 | 60 (47, 72) |
| 60+ | 502 | 8.33 ± 2.43 | 8 (7, 10) | 58.9 ± 14.6 | 61 (49, 70.75) |
| **Sex** |  |  |  |  |  |
| Female | 1682 | 8.20 ± 2.63 | 8 (7, 10) | 57.3 ± 14.7 | 59 (47.25, 69) |
| Male | 1135 | 7.33 ± 2.77 | 8 (5, 9) | 60.8 ± 14.5 | 64 (51, 73) |
| **Support** |  |  |  | **--** | **--** |
| Low (7-21) | 44 | 10.41 ± 3.51 | 10.5 (8, 12) | **--** | **--** |
| Low-Mod (22-35) | 177 | 9.06 ± 2.42 | 9 (8, 10) | **--** | **--** |
| Mod (36-49) | 522 | 8.19 ± 2.67 | 8 (7, 10) | **--** | **--** |
| Mod-High (50-64) | 863 | 7.86 ± 2.79 | 8 (6, 10) | **--** | **--** |
| High (65-77) | 1211 | 7.42 ± 2.57 | 8 (6, 9) | **--** | **--** |

**Figure A. Flow diagram**


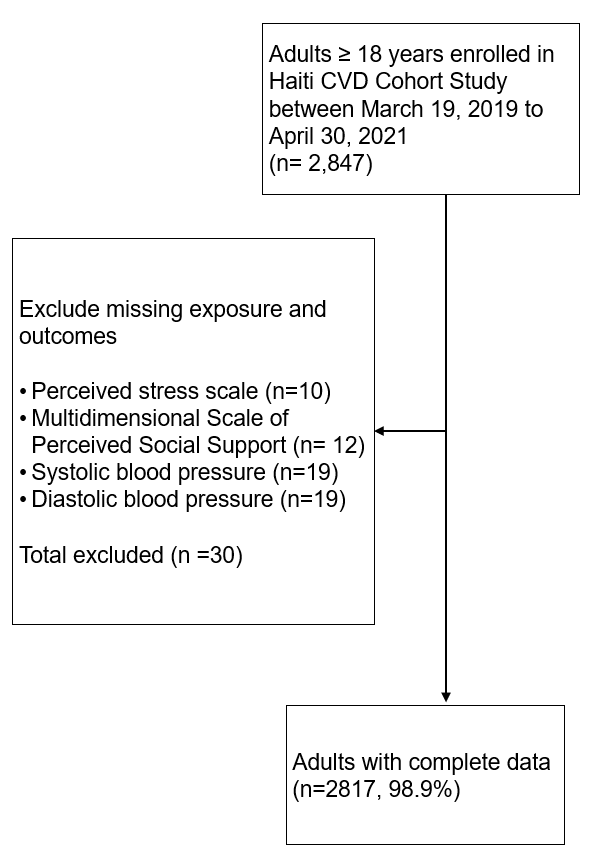


# STROBE Statement—Checklist of items that should be included in reports of *cross-sectional studies*

|  | Item No | Recommendation | Page No |
| --- | --- | --- | --- |
| **Title and abstract** | 1 | (*a*) Indicate the study’s design with a commonly used term in the title or the abstract | 1 |
|  |  | (*b*) Provide in the abstract an informative and balanced summary of what was done and what was found | 2-3 |
| Introduction | | | |
| Background/rationale | 2 | Explain the scientific background and rationale for the investigation being reported | 4 |
| Objectives | 3 | State specific objectives, including any prespecified hypotheses | 4-5 |
| Methods | | | |
| Study design | 4 | Present key elements of study design early in the paper | 5 |
| Setting | 5 | Describe the setting, locations, and relevant dates, including periods of recruitment, exposure, follow-up, and data collection | 5 |
| Participants | 6 | (*a*) Give the eligibility criteria, and the sources and methods of selection of participants | 5 |
| Variables | 7 | Clearly define all outcomes, exposures, predictors, potential confounders, and effect modifiers. Give diagnostic criteria, if applicable | 6 |
| Data sources/ measurement | 8* | For each variable of interest, give sources of data and details of methods of assessment (measurement). Describe comparability of assessment methods if there is more than one group | 5-6 |
| Bias | 9 | Describe any efforts to address potential sources of bias | 5-6 |
| Study size | 10 | Explain how the study size was arrived at | 5-6 |
| Quantitative variables | 11 | Explain how quantitative variables were handled in the analyses. If applicable, describe which groupings were chosen and why | 5-6 |
| Statistical methods | 12 | (*a*) Describe all statistical methods, including those used to control for confounding | 7 |
|  |  | (*b*) Describe any methods used to examine subgroups and interactions | 7 |
|  |  | (*c*) Explain how missing data were addressed | 7 |
|  |  | (*d*) If applicable, describe analytical methods taking account of sampling strategy | 7 |
|  |  | (*e*) Describe any sensitivity analyses | 7 |
| Results | | | |
| Participants | 13* | (a) Report numbers of individuals at each stage of study—eg numbers potentially eligible, examined for eligibility, confirmed eligible, included in the study, completing follow-up, and analysed | 8 |
|  |  | (b) Give reasons for non-participation at each stage | 6, 8 |
|  |  | (c) Consider use of a flow diagram | Fig S1 |
| Descriptive data | 14* | (a) Give characteristics of study participants (eg demographic, clinical, social) and information on exposures and potential confounders | 8-9 |
|  |  | (b) Indicate number of participants with missing data for each variable of interest | 8-9 |
| Outcome data | 15* | Report numbers of outcome events or summary measures | 8-9 |
| Main results | 16 | (*a*) Give unadjusted estimates and, if applicable, confounder-adjusted estimates and their precision (eg, 95% confidence interval). Make clear which confounders were adjusted for and why they were included | 8-9 |
|  |  | (*b*) Report category boundaries when continuous variables were categorized | 8-9 |
|  |  | (*c*) If relevant, consider translating estimates of relative risk into absolute risk for a meaningful time period | 8-9 |
| Other analyses | 17 | Report other analyses done—eg analyses of subgroups and interactions, and sensitivity analyses | 8-9 |
| Discussion | | | |
| Key results | 18 | Summarise key results with reference to study objectives | 9 |
| Limitations | 19 | Discuss limitations of the study, taking into account sources of potential bias or imprecision. Discuss both direction and magnitude of any potential bias | 12 |
| Interpretation | 20 | Give a cautious overall interpretation of results considering objectives, limitations, multiplicity of analyses, results from similar studies, and other relevant evidence | 9-13 |
| Generalisability | 21 | Discuss the generalisability (external validity) of the study results | 9-13 |
| Other information | | | |
| Funding | 22 | Give the source of funding and the role of the funders for the present study and, if applicable, for the original study on which the present article is based | online submission |
